# Effect of Virtual Reality on Cognitive Impairment and Clinical Symptoms among Patients with Schizophrenia in the Remission Stage: A Randomized Controlled Trial

**DOI:** 10.3390/brainsci12111572

**Published:** 2022-11-18

**Authors:** Shangda Li, Renchuan Liu, Bin Sun, Ning Wei, Zhe Shen, Yi Xu, Manli Huang

**Affiliations:** 1 Department of Psychiatry, The First Affiliated Hospital, Zhejiang University School of Medicine, Hangzhou 310003, China; 2 The Key Laboratory of Mental Disorder Management in Zhejiang Province, Hangzhou 310003, China; 3 Ningbo Psychiatric Hospital, 11 Rixingfang, Jiangbei District, Ningbo 315032, China; 4 Brain Research Institute of Zhejiang University, Hangzhou 310003, China; 5 Zhejiang Engineering Center for Mathematical Mental Health, Hangzhou 310003, China; 6 Xinyue Wellness Counseling and Consulting Services, LLC, Hangzhou 310000, China

**Keywords:** virtual reality, schizophrenia, MCCB, cognitive function

## Abstract

**Aims**: This intervention study evaluates the effect of a virtual reality cognition training system (VRCTS) on improving cognitive function and clinical symptoms in Han Chinese patients with schizophrenia in the remission stage. **Methods**: Sixty-eight patients with schizophrenia in the remission stage were recruited for this study and were randomly allocated to either the virtual reality training (VRT) group or the treatment-as-usual (TAU) group. For the VRT group, patients received training with the VRCTS for two weeks and antipsychotic treatment as usual, while the TAU group only received antipsychotic treatment as usual. Cognitive function and clinical symptoms before and after the two-week treatment were assessed by the MATRICS consensus cognitive battery (MCCB), positive and negative syndrome scale (PANSS), and personal and social performance scale (PSP). **Results**: The results showed that (1) VRCTS could improve MCCB composite scores and scores on 2 out of 7 cognitive domains: visual learning as well as reasoning and problem solving. It was also observed that (2) VRCTS could alleviate general psychopathology symptoms of PANSS, but did not exert effects on positive and negative symptoms among patients with schizophrenia in the remission stage. **Conclusions**: A therapeutic effect of VRCTS was observed in patients with schizophrenia in the remission stage. This may improve cognitive function and general psychopathological symptoms. Trial registration: China Clinical Trial Registry, ChiVTR1800016121.

## 1. Introduction

Schizophrenia is a complex, heterogeneous behavioral and cognitive syndrome that has a profound impact on the individual and society [1,2], with a global lifetime prevalence of 0.3–0.7% [3,4]. Patients with schizophrenia exhibit disrupted cognitive function, including reduced attention and memory, including working memory (WM) and episodic memory [5]. Schizophrenia in the remission stage is often defined as having mild or fewer symptoms assessed by clinical scales such as the brief psychiatric rating scale (BPRS), the scale for the assessment of positive symptoms (SAPS), the scale for the assessment of negative symptoms (SANS), or positive and negative syndrome scale (PANSS); furthermore, these symptoms, at least cognitive dysfunction, persist for a period of 6 months [6]. Since schizophrenia is a chronic mental disorder with a high disability rate, clinically, schizophrenia in many patients ultimately enters the remission stage.

Although symptoms such as hallucinations and delusions are alleviated in the remission stage, functional recovery usually does not occur. Cognitive function deficits sometimes remain in schizophrenia patients in the remission stage [7], and the disability associated with schizophrenia is largely due to cognitive impairment in this stage [8].

Currently, antipsychotics and second-generation antipsychotic drugs are the mainstay of treatment for schizophrenia and have a defined curative effect on positive, negative and cognitive symptoms [9]. However, the neurological side effects of antipsychotics hinder the efficacy of treating schizophrenia [10]. In addition, patient compliance with antipsychotics is often not satisfactory in clinical settings [11], especially while improvements in the positive symptoms are obtained. Furthermore, these drugs ineffectively alleviate cognitive impairment, such as working memory and attention [8]. Patients retain cognitive symptoms after long-term use of antipsychotics [12], and excessive doses of antipsychotics may induce poorer cognitive function [13]. Some studies have also shown that long-term use of antipsychotics might impair cognitive function [14].

Other studies have shown that psychological counseling, such as cognitive behavior therapy [15], cognitive remediation [16] and cognitive enhancement therapy [17], may be helpful for cognitive functioning in patients with schizophrenia, but these counseling therapies often require a therapist and an individualized treatment plan. In addition, patients may have problems understanding the therapy, and the therapeutic effects are sometimes delayed. All of these limitations might hinder the widespread application of counseling therapies. Therefore, a new adjunct treatment is urgently needed for lingering cognitive function impairments in patients with schizophrenia in the remission stage.

Virtual reality (VR) is an encouraging technology being applied in the treatment of psychiatric disorders. It produces interactive computer-generated environments that create a sensation of being in real worlds [18]. Many studies have investigated the effects of virtual reality training (VRT) in patients with schizophrenia. A study of 12 patients with schizophrenia reported that VRT significantly alleviated negative symptoms, social function, social anxiety and discomfort [19]. In addition, a significant improvement was observed in paranoid ideation and momentary anxiety in patients with psychotic disorders and paranoid ideation after VR-based cognitive behavioral treatment [20]. Freeman and colleagues showed that social environments and cognitive therapy based on VR showed significant reductions in delusional conviction in patients with persecutory delusion [21]. Another study also reported that VR therapy could help with self-esteem, anxiety symptoms and quality of life for patients with schizophrenia [22]. Regarding cognitive functioning, many studies have demonstrated that VR could mitigate cognitive function impairments in different disease conditions. Faria and colleagues reported that patients with stroke gained improvements in attention, memory, visuospatial abilities, global cognitive functioning and executive functions after 12 sessions of VR therapy [23]. Another study showed that after 24 sessions of VRT, poststroke patients improved in sustained attention [24]. VRT also exerted an enhanced effect on executive function, attention span and memory in older adults [25]. A previous study claimed that VRT could help with the rehabilitation of cognitive function. Wang and colleagues found that 10 days of VRT improved participants’ working memory and executive function [26]. In addition, a systematic review also showed that VR-based interventions may represent a novel and efficacious approach for improving cognitive and psychosocial functioning [27].

However, the literature is still lacking in knowledge on the effects of VRT for improving cognitive functioning in patients with schizophrenia in the remission stage, and few studies have focused on examining VRT in application to Han Chinese people. To resolve these issues, the present study designed a virtual reality cognition training system (VRCTS), which is relevant to Han Chinese people’s daily lives. We then evaluated the effect of the VRCTS on cognitive function impairment in patients with schizophrenia in the remission stage. We hypothesized that the VRCTS could attenuate the cognitive function impairments in patients with schizophrenia in the remission stage, and we proposed a model in which VR may have the potential to help shape brain health and simulate a daily life situation for rehabilitation.

## 2. Materials and Methods

This was a randomized controlled trial (RCT) with a parallel design. It was conducted in the First Affiliated Hospital of Zhejiang University School of Medicine and Ningbo Psychiatric Hospital from 1 June to 20 October 2019. The study was approved by the ethics committee of the First Affiliated Hospital of the Medical School of Zhejiang University (registration number: 2018533) in accordance with the Declaration of Helsinki, and was registered in the China Clinical Trial Registry under registration number ChiVTR1800016121.

### 2.1. Participants

Sixty-eight patients with schizophrenia were recruited from among the inpatients at Ningbo Psychiatric Hospital. Sun Bin, an experienced psychiatrist at Ningbo Psychiatric Hospital, was responsible for the recruitment of potential participants and screened all patients for eligibility. The inclusion criteria were as follows: aged 18 to 55; met the ICD-10 criteria for schizophrenia; were in remission stage; had positive and negative syndrome scale (PANSS) item scores of ≤3 for at least 6 months according to Andreasen’s criteria [6] (the PANSS items included delusions (P1), conceptual disorganization (P2), hallucinatory behavior (P3), blunted affect (N1), social withdrawal (N4), lack of spontaneity (N6), mannerisms/posturing (G5) and unusual thought content (G9)); had received atypical antipsychotics with a stable treatment dose for more than 1 month; and had normal vision and right-handedness. The exclusion criteria were as follows: a history of brain trauma, epilepsy and other neurological diseases or serious physical diseases; a diagnosis of a history of intellectual disability or a history of substance abuse in the past 30 days (except smoking); a history of electroconvulsive therapy in the past year; a history of using typical antipsychotics; pregnancy or a plan to become pregnant; and Wechsler adult intelligence scale-revised China (WAIS-RC) scores < 80. After providing a description of the study to the subjects, written informed consent was obtained. All participants then received scale assessments and the measurement and treatment research to improve cognition in schizophrenia (MATRICS) consensus cognitive battery (MCCB) at baseline (T0) and after two weeks of VRT (T1).

Patients fulfilling the inclusion criteria were randomly allocated to either the VRT group or the treatment-as-usual (TAU) group at a 1:1 ratio. The definition of the TAU group refers to the criteria of Olivier and colleagues [28]. For the VRT group, the patients received VRT for two weeks and antipsychotic treatment as usual, while the TAU group received only antipsychotic treatment as usual.

### 2.2. Cognitive Function Assessment

To assess cognitive functioning in patients with schizophrenia, some evaluation tools, such as the brief assessment of cognition in schizophrenia (BACS) and MCCB have been developed [29]. The MCCB is an accepted standard for measuring cognition in patients with schizophrenia and includes 10 different cognitive subtests. It has been recommended by the United States Food and Drug Administration (FDA) to assess cognitive impairment in schizophrenia [30,31]. It demonstrates excellent reliability and practicality. Recently, studies have reported that the MCCB is applicable for both individuals with first-episode schizophrenia and chronic schizophrenia [32], and investigations using the MCCB have focused on different cognitive domains in patients with schizophrenia.

In the present study, cognitive function was assessed by a trained psychiatrist using the MCCB at T0 and T1. The raters were blinded to the group assignment of the participants. The MCCB includes 10 neuropsychological tests clustered in 7 cognitive domains: speed of processing (SP), attention/vigilance (AV), working memory (WM), verbal learning (VeL), visual learning (ViL), reasoning and problem solving (RPS), and social cognition (SC) [33]. Each domain score was standardized to a T score derived using the MCCB computer scoring program (version 2.1.1, Psychological Assessment Resources, Inc., Lutz, FL, USA). Furthermore, the overall composite T score was calculated by averaging the standardized value of each test’s T score.

### 2.3. Clinical Symptom Assessment

Two scales, the PANSS and the personal and social performance scale (PSP), were used to evaluate the severity of clinical symptoms of patients with schizophrenia in the remission stage. All the assessments were conducted by Sun Bin, who was blinded to the group assignment.

### 2.4. VRT Procedure

VRT was executed once a day, five times per week, and lasted for two weeks. A supermarket situation based on VR techniques was designed. The virtual supermarket simulated a supermarket with a variety of goods, such as drinks, tea sets, kitchenware, fruits and vegetables. There was also a shopping cart. Unity 5.3.5f1 (Available online: https://unity3d.com, accessed on June 2017) and Visual Studio 2015 (Microsoft) were used to design and create the VR program. The details of the VR program were described in our previous study [34]. The patients were asked to complete different shopping tasks with different lists. The shopping tasks included task A and task B, and each task consisted of four levels. At the beginning of each task, the participants became familiar with the procedures as follows:

1. The patients learned to wear the helmet in a comfortable way, to enter the virtual supermarket and to use the joysticks to manipulate items in the virtual supermarket.
2. When a list of goods was presented on the screen, the patients read the list and closed it after memorizing the list.
3. The patients collected the goods presented on the list and put them in the shopping cart in the virtual supermarket using joysticks.
4. If the patients forgot the contents of the list, they could press the button on the joystick, and the list would be presented again.

**Figure 1:**
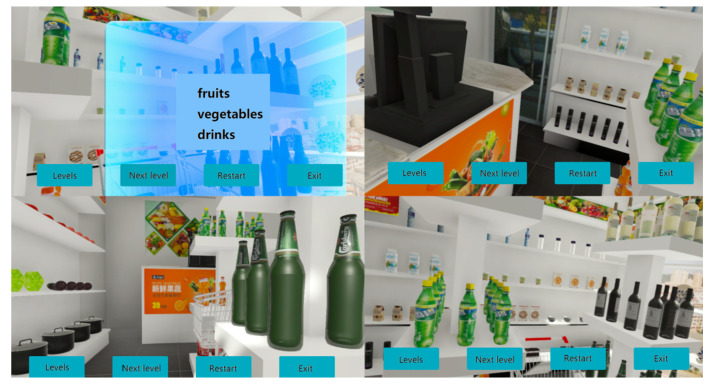
Participant perspective of the virtual reality supermarket.

A schematic describing the VRT is presented in Figure 1. The training included two kinds of tasks. Task A asked the patients to find goods in a certain category, such as fruits, vegetables and drinks, and put them into shopping carts. Task B instructed the participants to find specific goods, such as apples, tomatoes and cola. The number of goods ranged from 3 to 6 as the task level increased. As the number of goods increased, the working memory span needed was increased. Renchuan Liu administered the VRT procedures. To ensure consistency of treatment, our procedure asked the patients to finish the task in VRT from level 1 to level 4, and patients were not allowed to make arbitrary choices. If patients failed at one level, they would be asked to try this level again one more time; if they still failed, they would go on to the next level.

According to our previous study and Freeman’s report, the adverse events from VRT were usually mild and were not associated with the number of VR sessions or impact VR therapy [34,35]. We told patients once they felt uncomfortable, they could stop the task and tell us immediately.

### 2.5. Statistical Analysis

The data are expressed as the mean ± SD for the continuous variables.

The normality of the data distribution was judged by the Shapiro—Wilk test.

Baseline data, including age, sex, course of disease, and education year were analyzed for the comparability of baseline data. The chi-square test was used to compare the sexes of the two groups. Normally distributed data were expressed as the mean ± SD and analyzed by independent *t* tests, while nonnormally distributed data were expressed as the median (1st quartile, 3rd quartile) and analyzed by Mann—Whitney U tests.

Changes in T scores from the MCCB, PANSS and PSP at T0 and T1 in both groups were analyzed by Two-way repeated measures ANOVA. All statistical analyses were performed using SPSS version 19.0 (IBM, Chicago, IL, USA) for Windows.

## 3. Results

### 3.1. Demographic Characteristics and Baseline Data for the Two Groups

The total sample comprised 68 patients with schizophrenia. One patient in the TAU group was found to have adjusted his drug dosage in the previous month, so he did not receive the allocated intervention. Four of the patients in the VRT group were withdrawn because they could not finish the MCCB, and another patient in the TAU group was over age 55 at the beginning of the trial and so the intervention was discontinued. The other 30 patients in the VRT group and 32 patients in the TAU group completed the study. The final sample included 62 patients. One patient in each group felt dizzy during the VRT, but they finished the therapy because the dizziness was tolerable and disappeared after VRT had concluded. No other uncomfortable feelings or serious adverse events were reported. All patients finished the 10 VRTs.

There were no significant differences in age, sex, years of education, age of onset, or T scores from the MCCB, PANSS or PSP at baseline between the VRT group and TAU group (all *p* > 0.05). However, the course of disease for the patients in the TAU group (mean ± SD, 249.94 ± 97.55) was significantly longer than that of patients in the VRT group (195.10 ± 107.86) (Table 1).

The medication regimens of the two groups were as follows. The kind of medicine used by patients included antipsychotics, antidepressants, mood stabilizers, anxiolytics, and sedatives hypnotics. Olanzapine, clozapine, and risperidone were the most frequently used antipsychotics.

We used a chi-square test to evaluate the difference between the two groups, and the results showed no significant difference (value = 0.834, *p* = 0.934) (Table 2). We also listed the detailed medication regime of two groups as Supplementary Data (Table S1).

### 3.2. MCCB T Scores for the Two Groups

**Figure 2:**
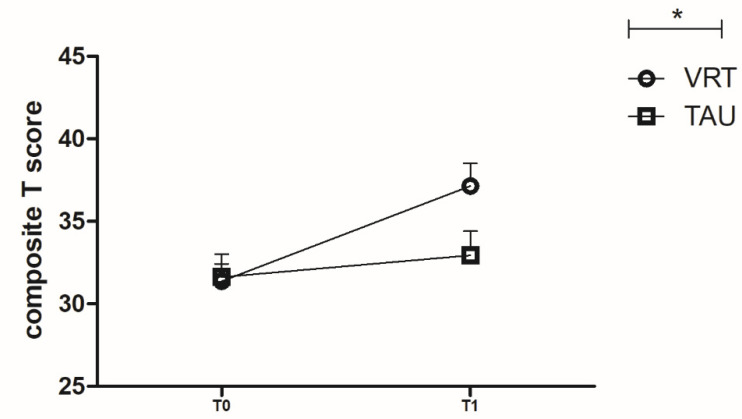
Legends: Figure 2 Line chart showing the changes of MCCB T scores between T0 (VRT: 31.34 ± 5.80; TAU: 31.61 ± 7.89) and T1 (VRT: 37.15 ± 7.54; TAU: 32.94 ± 8.28), *p* = 0.032, * *p* ≤ 0.05.

**Figure 3:**
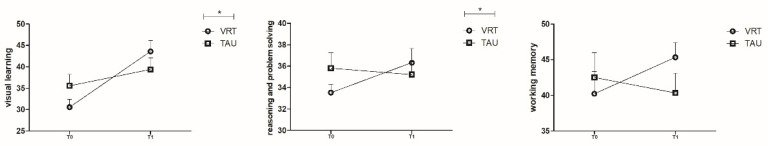
Legends: Figure 3 Line chart showing the changes of ViL, RPS and WM T scores between T0 (VRT: 30.57 ± 10.21, 30.57 ± 10.21 and 40.23 ± 17.08; TAU: 35.56 ± 15.19, 34 (30.25, 42)) and 42.50 ± 19.78 and T1 (VRT: 30.57 ± 10.21, 30.57 ± 10.21 and 45.33 ± 11.18; TAU: 35.56 ± 15.19, 34 (30.25, 42) and 40.34 ± 15.68), *p* = 0.002, 0.001, 0.086, respectively, * *p* ≤ 0.007).

**Figure 4:**
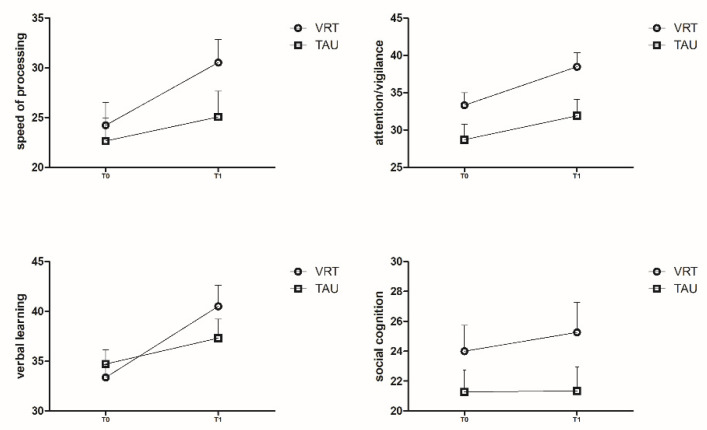
Legends: Figure 4 Line chart showing the changes of SP, AV, VeL, and SC T cores between T0 (VRT: 24.23 ± 12.46, 33.33 ± 9.18, 33.37 ± 7.14 and 24.00 ± 9.51; TAU: 22.66 ± 13.17, 28.72 ± 11.52, 35.56 ± 15.19 and 21.28 ± 8.38) and T1 (VRT: 24.23 ± 12.46, 38.47 ± 10.35, 40.50 ± 11.60 and 25.27 ± 11.09; TAU: 22.66 ± 13.17, 31.91 ± 12.74, 37.31 ± 10.82 and 21.34 ± 9.06), all *p* > 0.05).

As shown in Figure 2, Figure 3 and Figure 4, many changes in MCCB T scores were observed from T0 to T1 in the VRT group.

For the MCCB composite T score, two-way repeated measures ANOVA showed that there was a significant interaction between time and group (F = 19.119, *p* < 0.001). Pairwise comparisons showed that no difference was found in MCCB composite T scores between TAU and VRT group at T0 (F = 0.00, *p* = 0.985). However, the MCCB composite T score of VRT group was significantly higher than that of the TAU group at T1 ((37.15 ± 7.54 vs. 32.94 ± 8.28; F = 5.093, *p* = 0.032, η^2^ = 0.149). In VRT group MCCB composite T score at T1 was significantly higher than that at T0 (F = 68.630, *p* < 0.001, η^2^ = 0.703) while in TAU group no difference was found between T0 and T1(F = 2.694, *p* = 0.111) (Figure 2).

We also compared the result of seven cognitive domains from T0 to T1 I between two groups, with a significance level of 0.05/7, Bonferroni correction.

For RPS T score, two-way repeated measures ANOVA showed that there was a significant interaction between time and group (F = 8.441, *p* = 0.007); pairwise comparisons showed that no difference was found in RPS T score between TAU and VRT group neither, at T0 nor at T1 (both *p* > 0.007). However, in the VRT group RPS the T score at T1 was significantly higher than that found at T0 (F = 11.353, *p* = 0.002, η^2^ = 0.281), while in the TAU group no difference was found between T0 and T1 (F = 0.407, *p* = 0.528) (Figure 3).

For ViL T score, two-way repeated measures ANOVA showed that there was a significant interaction between time and group (F = 10.245, *p* = 0.003); pairwise comparisons showed that no difference was found in ViL T score between TAU and VRT group, neither at T0 nor at T1(both *p* > 0.007). However, in the VRT group, an RPS T score at T1 was significantly higher than T0 (F = 30.176, *p* < 0.001, η^2^ = 0.510), while in the TAU group no difference was found between T0 and T1(F = 2.958, *p* = 0.095) (Figure 3).

For the WM T score, two-way repeated measures ANOVA showed that there was no interaction between time and group (F = 3.561, *p* > 0.007);

In addition, VRT had no significant effect on the WM T score (F = 0.594, *p* = 465). Nor was significant difference found in the WM T score at different time points (F = 0.375, *p* > 0.545) (Figure 3).

For the SC T score, two-way repeated measures ANOVA showed that there was no interaction between time and group (F = 0.479, *p* = 0.495).

In addition, VRT had no significant effect on the SC T score (F = 1.976, *p* = 0.170). Nor was a significant difference found in the SC T score at different time points (F = 1.735, *p* = 0.198) (Figure 4).

For the VeL T score, two-way repeated measures ANOVA showed that there was no interaction between time and group (F = 3.009, *p* = 0.093).

In addition, VRT had no significant effect on the VeL T score (F = 0.340, *p* = 0.564). However, significant difference was found in VeL T score at different time points (F = 33.802, *p* < 0.001, η^2^ = 0.538) (Figure 4).

For AV T score, two-way repeated measures ANOVA showed that there was no interaction between time and group (F = 0.305, *p* = 0.585).

In addition, VRT had no effect on the AV T score (F = 3.183, *p* = 0.085.) A significant difference was found in AV T score at different time points (F = 26.620, *p* < 0.001, η^2^ = 0.479) (Figure 4).

For SP T score, two-way repeated measures ANOVA showed that there was no significant interaction between time and group (F = 4.337, *p* = 0.046);

In addition, VRT had no significant effect on the SP T score (F = 1.211, *p* = 0.280). A significant difference was found in SP T score at different time points (F = 27.975, *p* < 0.001, η^2^ = 0.491) (Figure 4).

### 3.3. Clinical Symptoms Assessment in the Two Groups

PANSS scores for the two groups

For PANSS total score, two-way repeated measures ANOVA showed that there was no interaction between time and group (F = 1.437, *p* = 0.258).

In addition, VRT had potential effect on the PANSS total score, with no significance (F = 4.909, *p* = 0.051). Significant difference was found of PANSS total score at different time points (F = 11.044, *p* = 0.008) (Table 3).

We also compared the PANSS P, N and G scores from T0 to T1 I between two groups, with a significance level of 0.05/3, Bonferroni correction.

For PANSS P score, two-way repeated measures ANOVA showed that there was no interaction between time and group (F = 1.786, *p* = 0.211).

In addition, VRT had no significant effect on the PANSS P score (F = 0.217, *p* = 0.651). Neither significant difference was found of PANSS P score at different time points (F = 4.224, *p* = 0.067) (Table 3).

For PANSS N score, two-way repeated measures ANOVA showed that there was no interaction between time and group (F = 0.426, *p* = 0.529).

In addition, VRT had potential effect on the PANSS N score, with no significance (F = 4.290, *p* = 0.065). Neither significant difference was found in PANSS N score at different time points (F = 0.213, *p* = 0.654) (Table 3).

For PANSS G score, Two-way repeated measures ANOVA showed that there was no interaction between time and group (F = 3.893, *p* = 0.077).

In addition, VRT had significant effect on the PANSS G score (F = 8.405, P = 0.016, η^2^ = 0.458). No difference was found in PANSS G score at different time points (F = 0.111, *p* = 0.764) (Table 3).

To explore which general psychopathology symptoms were improved after VRT, we compared general psychopathology symptoms scores from G1-G16 at baseline by independent *t* test, and no significant difference was found (all *p* > 0.05). Then, we compared each PANSS G score from G1 to G16. The results showed that the disturbance of volition (G13) at T1 in the VRT group were significantly lower than TAU group (F = 7.335, *p* = 0.014, η^2^ = 0.268. In addition, lack of judgment and insight (G12) and poor attention (G11) at T1 in the VRT group was lower than in the TAU group, but the difference was not significant. (F = 4.943, *p* = 0.038, η^2^ = 0.198; F = 3.727, *p* = 0.068, η^2^ = 0.157., respectively).

PSP scores for the two groups.

For PSP score, two-way repeated measures ANOVA showed that there was no interaction between time and group (F = 0.079, *p* = 0.785).

In addition, VRT had no significant effect on the PSP score (F = 2.404, *p* = 0.125). A significant difference was found in PSP score at different time points (F = 25.793, *p* = 0.001) (Table 3).

## 4. Discussion

The dilemma is that patients with schizophrenia in the remission stage always suffer from lingering cognitive impairment. VRT is a potential tool for cognitive rehabilitation. A previous study showed that cognitive function improved from treatment with VRT, but research focused on schizophrenia has been limited. Ten sessions of exposure to the VR program were described as an emerging method for significant gains in cognitive function in patients with schizophrenia [36]; however, the participants in this study were all older patients, and the VR program was also different from the supermarket situation. The present study designed the VRCTS, which offered a virtual environment closely related to people’s daily lives. It was an immersive virtual supermarket that easily generated a sense of being in a real supermarket, and the participants experienced interest and enjoyed the process of tasks, such as choosing goods, in the supermarket. The present study found that 10 sessions of VRCTS mitigated cognitive impairment in patients with schizophrenia in the remission stage. To our pleasant surprise, the results of the PANSS showed that general psychopathology symptoms were also alleviated after exposure to the VRCTS.

The results showed that MCCB composite scores, scores in two cognitive domains, including ViL and RPS, were all significantly improved after 10 sessions of the VRCTS.

This finding is consistent with a previous study that showed that forty patients with schizophrenia achieved significant improvements in the MCCB domains SP, AV, WM, VeL and RPS after ten sessions of the VR game program [37]. The differences in MCCB domains that improved in the two studies may be related to the different tasks in the virtual situations. In the present study, to complete the task in a virtual situation, the participants needed to receive instructions from the screen, remember relative information, find and recognize the goods in the virtual supermarket and then put them into a shopping cart. This process involved domains of cognitive function such as information acquisition, visual stimulation, memorization, cued-recall, and problem solving, which are relative to SP, WM, ViL and RPS. When patients execute the tasks, the cognition mentioned above is enhanced. In addition, while the VRT task level increased, the number of goods increased, and the working memory span needed increased. As a result, while patients completed tasks and repeated training in the virtual situation, their cognitive functioning also improved at the same time.

Regarding the clinical symptoms of patients, VRT has been used in the rehabilitation of schizophrenia and has alleviated symptoms. A study showed that overall clinical symptoms were significantly reduced in patients with schizophrenia after 10 sessions of VR training [38]. Social function also improved after patients received VR interview training [39]. However, in our study, we found that VRT only improved the general psychopathological symptoms in patients, rather than the positive symptoms or negative symptoms. This difference might be related to the design of the VR situation. Our VRCTS task simulated a daily life situation, namely, shopping in a supermarket; it might not mitigate positive and negative symptoms of schizophrenia but exerts an effect on general symptom rehabilitation. The results showed that poor attention, judgment and insight, and disturbance of volition were improved after VRT, and all of them were involved during VRT. In VR situations, patients receive rehabilitation training that involves situations that reflect their daily life, such as shopping, which is helpful for attention and volitional action. As some antipsychotics with long-term use induce some aspects of metabolic syndrome or other side effects, [40] we suggest that VRCTS may be a better choice for residual general psychopathology symptoms, such as attention, judgment and volition, which may be more relevant to VRT.

In regard to social function, a previous study found that the PSP scores of patients with schizophrenia improved after rehabilitation training based on VR [41]. However, the present study found that the VRCTS did not help improve personal and social function. This may be related to the duration of therapy. The VR therapy in the previous study lasted 8 weeks, but in the present study, treatment lasted for only two weeks. More research is needed to explore the effect of the VRCTS on improving social function.

In addition, it is worth mentioning that the side effects of VRT seemed minor; only 2 out of 62 participants felt a mild level of discomfort during VRT, and they were able to finish the treatment. The feedback from patients regarding the VRCTS was satisfactory. The staff asked each patient to evaluate the VRCTS after they completed VRCTS training: no patient felt dissatisfied, and some thought the tasks in the virtual situations were challenging and attractive. This may be related to the interactive therapeutic environment created by VR technology [18].

Although cognitive remediation and rehabilitation are essential components of care for people with schizophrenia [42], resources for rehabilitation services are still insufficient. As the VRCTS could partly ameliorate cognitive function and the acceptability of patients in this study was high, it may be a prospective treatment in a rehabilitation context for patients with schizophrenia in the remission stage, and if the VRCTS was widely used, it might overcome the bottleneck in the provision of rehabilitative services in China.

Nevertheless, the current trial had several limitations. First, the sample size of participants was small. Second, although we have elaborated the kind of medicine in the two groups, the patients received different types of second-generation antipsychotics, which may have interfered with their cognitive function and clinical symptoms. Furthermore, the two groups were not matched for the course of disease, which influenced cognitive function in a previous research. MCCB scores were similar between two groups at baseline in present study so the influence of course of disease can be excluded [43]. Another shortcoming was that our study lacked follow-up after the two groups finished the intervention, so we could not identify the long-term effects of VRT. We also lack data from drug urinalysis, lumbar puncture, electroencephalogram and brain magnetic resonance imaging for patients with schizophrenia, which may reveal the mechanism of VRT. As a result, more studies involving larger sample sizes and longer follow-up times are needed in the future.

## 5. Conclusions

In conclusion, we found that VRCTS had therapeutic effects for patients with schizophrenia in the remission stage. The VRCTS may improve cognitive function impairment and general psychopathological symptoms. The present study suggests that the VRCTS is a promising adjunct intervention for the rehabilitation of patients with schizophrenia in the remission stage.

## Figures and Tables

**Table 1:** Demographic characteristics and baseline data of the two groups.

	VRT Group (n = 30)	TAU Group (n = 32)	*p*
Age	46 (37, 50)	47.5 (37.25, 51.75)	0.178 ^b^
Sex (male/female)	20/10	19/13	0.606 ^c^
Course of disease (month)	195.10 ± 107.86	249.94 ± 97.55	0.040 ^a^
Educational years	10.5 (9, 12)	9.5 (9, 12)	0.673 ^b^
Age of onset	22.5 (19, 28.25)	22 (18.25, 27.75)	0.389 ^b^
MCCB			
SP	24.23 ± 12.46	22.66 ± 13.17	0.630 ^a^
AV	33.33 ± 9.18	28.72 ± 11.52	0.088 ^a^
WM	40.23 ± 17.08	42.50 ± 19.78	0.632 ^a^
VERL	33.37 ± 7.14	34.72 ± 8.02	0.487 ^a^
VIL	30.57 ± 10.21	35.56 ± 15.19	0.136 ^a^
RPS	33.5 (31, 38)	34 (30.25, 42)	0.344 ^b^
SC	24.00 ± 9.51	21.28 ± 8.38	0.236 ^a^
Composite score	31.34 ± 5.80	31.61 ± 7.89	0.882 ^a^
PANSS			
PANSS total	43 (38, 48)	42 (39, 48.50)	0.767 ^b^
PANSS P	7 (7, 9)	7 (7, 9)	0.944 ^b^
PANSS N	11 (9.25, 13)	12 (11, 14)	0.064 ^b^
PANSS G	19 (18, 23)	19 (17.5, 21)	0.709 ^b^
PANSS P1	1.46 ± 0.88	1.21 ± 0.58	0.401 ^a^
PANSS P2	1.00 ± 0.00	1.35 ± 0.74	0.097 ^a^
PANSS P3	1.15 ± 0.55	1.21 ± 0.58	0.784 ^a^
PANSS N1	1.55 ± 0.78	1.86 ± 0.67	0.264 ^a^
PANSS N4	1.61 ± 0.77	2.00 ± 0.55	0.153 ^a^
PANSS N6	1.31 ± 0.48	1.43 ± 0.65	0.589 ^a^
PANSS G5	1.08 ± 0.28	1.00 ± 0.00	0.309 ^a^
PANSS G9	1.21 ± 0.43	1.08 ± 0.28	0.334 ^a^
PSP	70.82 ± 8.34	67.08 ± 5.71	0.239 ^a^

Note: ^a^ independent *t* test, ^b^ Mann—Whitney U test, ^c^ Chi-square test (VRT N = 30; TAU N = 32) PANSS items for defining remission stage according to Andreasen’s criteria: delusions (P1), conceptual disorganization (P2), hallucinatory behavior (P3), blunted affect (N1), social withdrawal (N4), lack of spontaneity (N6), mannerisms/posturing (G5) and unusual thought content (G9). Abbreviation: VRT: virtual reality therapy; TAU: treatment-as-usual; MCCB: MATRICS Consensus Cognitive Battery; SP: speed of processing; AV: attention-vigilance; WM: working memory; VeL: verbal learning; ViL: visual learning; RPS: reasoning/problem solving; SC: social cognition; PNASS: Positive and Negative Syndrome Scale, PANSS G: PANSS general psychopathology, PANSS P: PANSS positive; PANSS N: PANSS negative; PSP: Personal and Social Performance Scale.

**Table 2:** The medication regime of two groups.

Medicine to Use	Number of Patients in VR Group	Number of Patients in TAU Group	Chi-Square Test
Atypical antipsychotics	30	32	Value = 0.834
Antidepressants	8	7	*p* = 0.934
Mood stabilizers	8	12	
Anxiolytics	2	2	
Sedative-hypnotics	5	7	

Abbreviation: VRT: virtual reality therapy; TAU: treatment-as-usual.

**Table 3:** Clinical symptoms before and after intervention in the two groups.

	VRT Group		TAU Group		F	P
	T0	T1	T0	T1		
PANSS						
PANSS total	43 (38, 48)	37 (31, 42)	42 (39, 48.50)	39 (35, 41.50)	1.964	0.051
PANSS P	7 (7, 9)	7 (7, 8)	7 (7, 9)	7 (7, 9)	3.149	0.651
PANSS N	11 (9.25, 13)	10 (7, 14)	12 (11, 14)	12.5 (11, 14.5)	4.290	0.065
PANSS G	19 (18, 23)	17 (16, 21)	19 (17.5, 21)	19 (17.25, 20.75)	4.450	0.016 *
PSP	70.82 ± 8.34	72.36 ± 8.15	67.08 ± 5.71	68.25 ± 5.41	0.297	0.603

Note: (VRT N = 30; TAU N = 32) T0: baseline; T1: past two weeks of VRT. Abbreviations: VRT: virtual reality training; TAU: treatment-as-usual; PNASS: Positive and Negative Syndrome Scale, PANSS G: PANSS general psychopathology, PANSS P: PANSS positive; PANSS N: PANSS negative; PSP: Personal and Social Performance Scale. * *p* ≤ 0.017.

## Data Availability

The data presented in this study are available on request from the corresponding author. The data are not publicly available due to privacy of patients with schizophrenia.

## Supplementary Materials

The following supporting information can be downloaded at: https://www.mdpi.com/article/10.3390/brainsci12111572/s1, Table S1: The detailed medication regime of two groups.
