# Supplementary material for: Effect of Virtual Reality on Cognitive Impairment and Clinical Symptoms among Patients with Schizophrenia in the Remission Stage: A Randomized Controlled Trial
Source: Brain Sci. 2022 Nov 18;12(11):1572. doi: 10.3390/brainsci12111572 (PMC9688710; doi:10.3390/brainsci12111572)
Supplement: Supplementary file 1 [file brainsci-12-01572-s001.zip › brainsci-1925410-supplementary.pdf]

supplementary data

Table S1. The detailed medication regime of two groups.

Medicine to use	number of Patients in VR group	number of Patients in TAU group
Risperidone	8	6
clozapine	8	9
amisulpride	3	5
Quetiapine	3	4
Olanzapine	6	7
Ziprasidone	3	3
Aripiprazole	3	5
paliperidone palmitate	2	1
Sertraline	2	3
Venlafaxine	1	1
Duloxetine	1	1
escitalopram	1	1
Paroxetine	3	1
Sodium Valproate	5	6
lithium carbonate	3	6
Tandospirone	2	1
Buspirone	0	1
Alprazolam	2	0
Zopiclone	3	1
oxazepam	0	2
Lorazepam	0	4

Abbreviation: VRT: virtual reality therapy; TAU: treatment-as-usual.
